# The Australian National Pollutant Inventory Fails to Fulfil Its Legislated Goals

**DOI:** 10.3390/ijerph14050478

**Published:** 2017-05-04

**Authors:** Nathan Cooper, Donna Green, Katrin J. Meissner

**Affiliations:** 1 Climate Change Research Centre, University of New South Wales, Kensington, Sydney 2052, Australia; donna.green@unsw.edu.au (D.G.); k.meissner@unsw.edu.au (K.J.M.); 2 Australian Research Council’s (ARC) Centre of Excellence for Climate System Science, Canberra 2601, Australia

**Keywords:** pollutant release and transfer registers, Australia, lead, national pollutant inventory, airborne emissions, Mount Isa

## Abstract

Publically accessible pollution databases, such as the Australian National Pollutant Inventory, contain information on chemical emissions released by industrial facility and diffuse sources. They are meant to enable public scrutiny of industrial activity, which in turn, is meant to lead to industries reducing their pollution. In Australia, however, concerns have been consistently raised that this process is not occurring. To assess whether Australia’s National Pollutant Inventory is fulfilling its legislated goals, we examined the accuracy and consistency of the largest facility and diffuse source of airborne lead, a major pollutant of concern for public health. Our analysis found that the emissions estimates provided by the Inventory were not accurate and were not consistent with other sources of emissions within the Inventory, potentially distorting any user interpretation of emissions estimates provided by the National Pollutant Inventory. We conclude that for at least these important public health pollution sources, the Inventory does not fulfil its legislated goals.

## 1. Introduction

The National Pollutant Inventory (NPI) is an Australian database that lists the emissions and waste transfers of harmful chemicals that occur over a specified threshold from industrial and diffuse sources [1,2,3]. In 1998, the NPI’s function and goals were formally legislated through a National Environment Protection Measure (NEPM) [4]. The goals as listed in Clause 6 were to:

“(a) collect a broad base of information on emissions and transfers of substances on the reporting list, and (b) disseminate the information collected to all sectors of the community in a useful, accessible and understandable form”.

[1] (p. 12)

The goals set within the NEPM were developed with the intention of achieving broader aims related to environmental sustainability and community knowledge [1,5,6]. These broader aims were to inform the public and policy-makers on the quantities and geographic locations of toxic emissions; and, in so doing, to reduce industrial pollution through public scrutiny [1,2,5,7].

The goals and aims of the NPI were informed by domestic concerns about industrial pollution as well as international obligations [6,8]. Prior to the creation of the NPI, there was public concern regarding health and environmental risks that could occur from industrial pollution, and as a consequence, greater information and accountability was sought [8,9]. The 1992 United Nations Conference on Environment and Development, and the subsequent establishment of Agenda 21, highlighted the need for standardised, environmental pollution databases; now known as Pollutant Release and Transfer Registers (PRTR) [2,10]. The need for citizens to have access to information on environmentally hazardous pollutants, which later became known as the community “right-to-know” were also first established at these international governmental conferences [2] (p. 12) [10] (p. 21) [11]. Shortly after, the Organisation of Economic Co-operation and Development released a recommendation strongly encouraging its member countries to create national environmental pollutant databases [11,12].

Australia announced the decision to create a PRTR in 1992. This was followed by the Australian Government’s Commonwealth Environment Protection Agency releasing a Public Discussion Paper, outlining some of its goals in 1994 [4,7]. This document referred to the need to satisfy international environmental pollution monitoring and reporting obligations, as well as discussing the broader aims of the NPI to satisfy community right-to-know, and to promote reduced waste and cleaner forms of production. 

Since its inception, numerous concerns from various stakeholders have been raised regarding the functionality of the NPI [2,4,5]. Some of these queries directly relate to the NPI’s ability to fulfil its stated goals. 

One concern relates to the accuracy of the pollution estimates that are provided by industry, State, and Territory governments, since these pollution emissions are derived from estimates rather than direct measurement. Emissions estimates are significantly affected by the estimation method used, and the consistency of the temporal and spatial application of consistent methods, if any meaningful trend or comparison analysis is to be carried out [5,13,14]. This concern relates directly to the goals of the NPI. The NPI’s first goal “to collect a broad base of information on emissions…” suggests that information should be collected from various sources using varied methods to enable the release of the best information to users. Despite this, when collecting data for emissions from a particular listed source, the NPI only collects data submitted by the source, which submits a single figure for their emissions each time. As there is an absence of other data collected for emissions from a source, it is necessary that each estimate is accurate to be useful to users.

A second concern relates to the issue of how non-scientific users “interpret” the emissions estimation figures. Industry representatives have voiced their concern that the data are open to misinterpretation by community and environmental groups due to a lack of explanatory information available on the NPI website to enable non-specialist users to understand what their searches were returning to them [6,15]. This concern relates directly to the NPI’s goal to provide emissions information in a “useful, accessible and understandable form” to “all sectors of the community”, including non-scientifically trained public users and policy-makers [1] (p. 12) [2].

If data provided by the NPI are to be useful to non-scientifically trained users, it must maintain an accurate comparison between all sources. Furthermore, to allow temporal comparison, past emissions estimates and current estimates for a source must be carried out in a manner that allows comparison [6,13]. Because State and Territory governments are responsible for assessing industry emissions estimates and determining diffuse source emissions for their jurisdiction, any varying standards and methods of estimation between the governments will complicate attempts to compare emissions. This situation increases the likelihood of reporting inconsistency across NPI data for location and time [5]. 

If the ability of the NPI to achieve its goals is compromised, this is likely to have significant impacts for its ability to achieve its broader aims. The success or failure of the NPI to achieve its goals and aims could in turn lead to real consequences to local communities, as health and environmental risks may be either revealed to (or hidden from) the public and policy-makers—for whom the NPI was created to empower. For these reasons, the analysis undertaken by this paper seeks to assess how well the NPI fulfils its goals, specifically by assessing: the accuracy of emissions estimates from the NPI; and the consistency of emissions estimates between sources, as well as between current and past estimates, to enable an assessment of user interpretability. 

Furthermore, with the scheduled review of the NPI in 2017 [16] there is a real and timely opportunity to provide quantitative assessment of the NPI’s original goals and aims—something that has yet to be reported in the peer-reviewed literature to date.

## 2. Methods

To test if the NPI is achieving its stated goals, we selected a single chemical from the NPI list to examine in detail. We selected a chemical that was: emitted nationally from facility and diffuse sources; has historic, as well as current, emissions; and that has widely-known, and well-documented, human health effects. Based on these criteria, we selected “Lead and compounds” (hereafter referred to as lead) as it fit all these categories. We decided to focus on airborne lead emissions, rather than emissions to land or water, because for most categories of toxins this is the largest emissions pathway, and because the impact of the pollutant is strongly related to the distance from a pollution source. For this reason, the accuracy of airborne emissions is of particular interest to the general public.

We examined the largest facility and diffuse sources of airborne lead emissions separately for the period between July 2013 and June 2014 (as NPI estimates are calculated for each financial year, this was the most recent full year of data available at the time of the analysis). The largest facility and diffuse sources were found by searching for the chemical “Lead and compounds” in the “Search by Form” tab of the online NPI database. After carrying out this step, the largest identified facility source for lead was emitted from Mt Isa Mines, situated in North West Queensland (hereafter referred to as MIM), listed in the category of basic non-ferrous smelter. The largest diffuse source for lead was located in the Pilbara and Bunbury airsheds, in Western Australia (WA) listed in the category of paved/unpaved roads [17] (see Supplementary Materials Figure S1). 

### 2.1. Facility Source Emissions

#### 2.1.1. Assessment of the Accuracy of NPI Facility Emissions Estimations

Annual emissions estimates for MIM and other facility sources have been collected and published by the NPI for every financial year since the NPI’s opening [17]. To test the accuracy of the MIM lead emission estimates in the NPI, we compared the changes in MIM lead emissions estimates from past years with yearly changes in observed ambient airborne lead concentrations obtained from the Queensland Government. This method emulates Marchi and Hamilton’s approach [18], which relies on there being only one major source of emissions in an area [18]. The location of MIM satisfied this requirement because it is the most significant source of emissions of lead in air in the Mt Isa region [19,20]. Moreover, there have been concerns raised regarding high blood lead levels of residents in Mt Isa, demonstrating the need for accurate emissions estimates to inform residents of potential health risks [19,21].

This form of analysis assumes that the emissions released from a source, and the ambient air concentration near that source, will be strongly correlated [18]. This analysis is useful even though meteorological factors can affect airborne emissions [19,20,22]. Although annual averages of ambient concentrations will be influenced by prevailing wind conditions and inter-annual variability, we tested whether short term meteorological factors will affect annual averages significantly. We did not find that they significantly influenced the results of our analysis.

The airborne lead emissions estimation data obtained from the NPI were compared to annual observed ambient lead concentration data. The ambient airborne lead dataset was obtained by request from the Queensland Government’s Department of Environment and Heritage Protection (EHP), which publishes the annual averages in their monthly reports for North Queensland [23]. EHP measures the average micrograms per cubic metre of lead in air once every six days over a 24 h period (µg/m³/day), and is available in its complete form from 2009. Measurements are taken from ‘the Gap’, a station in the centre of Mt Isa (see Supplementary Materials Figure S2). From these measurements, annual average values for EHP measurements for every year between July 2009–June 2010 and July 2013–June 2014 were estimated. Due to a number of values below a detection limit, we were unable to conclusively calculate each yearly average outright, as substituting the zero-values (as the EHP does) or the detection limit would under or over-estimate the annual average respectively. To account for this issue, several different measures, each with their own limitations, were used to estimate yearly averages. These were: the Kaplan-Meier (K-M) method, Lognormal robust Regression on Order Statistics (LROS), the maximum possible value, and the minimum possible value (see Appendix A for more details). 

A second observational dataset from EHP that was considered for assessment was the hourly ambient lead data, which are published in near real-time online [24]. Although the hourly data covered the same time period more extensively than the original dataset, the data were not validated and therefore could not be used in our analysis. 

#### 2.1.2. Assessment of the Consistency of NPI Facility Emissions Estimations

(a) We examined the consistency of methods used by MIM to estimate and report emissions to the NPI over time. This approach was taken because the NPI recommends consistency in the methodological technique so that an accurate picture of the trends over time can be developed [13]. This analysis was supplemented with emissions estimates from the NPI for lead emissions from MIM for every year between July 2001–June 2002 and July 2013–June 2014 [17]. This dataset was examined to determine if there was a distinctive correlation between method changes and emissions estimates from MIM (see Appendix B for more details).

(b) We compared the estimation methods used by MIM for point source and fugitive emissions to those used by the Nystar smelter in Port Pirie, South Australia (hereafter referred to as “Port Pirie smelter”) and the Perilya Broken Hill mine, New South Wales (hereafter referred to as “Broken Hill Mine”) for every year between July 2001-June 2002 and July 2013-June 2014 to determine the consistency of estimation methods between similar sources. This analysis was supplemented with additional data to assess the influence of method changes on emissions estimates over time, relative to changes in sources of emissions [17]. Lead emissions estimates from the NPI for every year from July 2001–June 2002 to July 2013–June 2014 for all three sources were examined to determine whether there was a distinctive correlation between method changes and their emissions estimates.

### 2.2. Diffuse Source Emissions

#### 2.2.1. Assessment of Accuracy for Diffuse Source Emissions 

Our analysis of accuracy for diffuse emissions provided by the NPI examined the methods used to estimate emissions from paved/unpaved roads in the Pilbara and Bunbury airsheds, Western Australia (WA). We examined the methods of the NPI’s estimation of diffuse source emissions because currently there are no lead-in-air monitoring stations in WA, preventing comparisons with observational data [25]. 

As methods of estimating emissions from paved/unpaved roads were originally derived external to the NPI, we assessed the accuracy of these methods. We then examined whether there are any updates in the external methods that have not been incorporated into the current online NPI Emissions Estimation Techniques Manuel (EETM). Finally, we examined previous research conducted on the accuracy of these external methods. This approach was taken to reveal if there were any shortcomings in the external methods that were either not updated since publication in the NPI, or, were still present in their updated versions.

#### 2.2.2. Assessment of Consistency for Diffuse Source Emissions 

To assess the consistency of airborne lead data from paved/unpaved roads in the Pilbara and Bunbury airsheds, WA, we compared the magnitude of these emissions estimates to those from paved/unpaved roads in other States’ airsheds. We then compared estimation methods to explore the methodological differences and identify any potential causes of variation.

Finally, we examined what year diffuse airshed emissions estimates from the Pilbara and Bunbury airsheds and other States’ represented. This is determined by the data that are used to calculate an estimation, and not the year that the estimate is calculated. For example, an estimate for the annual emissions from an airshed may be based off data measured in 2003, therefore the estimate is representative of emissions released in 2003, even if the calculations are made later. This step was important because diffuse estimations are not performed annually and, therefore, there may be potentially significant variation in what year emissions estimates represent. 

## 3. Results

### 3.1. Facility Source Emissions

#### 3.1.1. Accuracy of NPI Emissions Estimations

**Figure 1:**
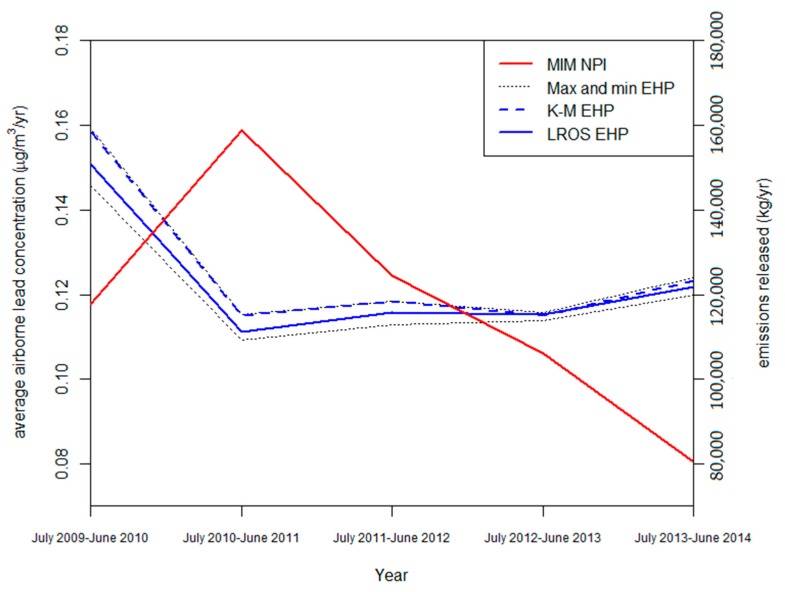
Comparison of annually averaged lead concentration using various analysis techniques calculated from Environment and Heritage Protection (EHP) data and The National Pollutant Inventory (NPI) annual emission estimates.

There were significant differences in the year-to-year changes between the EHP data and NPI emission estimates over the entire time period. As would be expected, changes in the average lead concentrations measured by the EHP were relatively consistent across all analysis techniques. Figure 1 shows that EHP concentration measurements decreased significantly from the year July 2009–June 2010 to July 2010–June 2011, in contrast to the sharp peak in NPI estimates over this time. Furthermore, NPI estimates decreased significantly each year between July 2010–June 2011 and July 2013–June 2014, contrasting with the relatively consistent EHP concentration measurements over this time. These results indicate significant discrepancies between NPI estimated emissions and EHP observational data over this entire time period.

#### 3.1.2. Consistency of NPI Emissions Estimations

(a) The NPI reports that MIM has changed its point emission estimation method seven times in 14 years, with no particular method of estimation used consistently over that time. These results demonstrate that point source emissions estimates from MIM are not consistent over time, suggesting that emissions estimates from different years cannot be compared with accuracy. By contrast, fugitive emission estimation methods changed four times between the years of July 2001–June 2002 and July 2013–June 2014. Furthermore, MIM consistently used one particular method for fugitive emissions, only adopting additional methods between the years of July 2007–June 2008 and July 2010–June 2011. These results appear to show that estimations for fugitive emissions from MIM concur with NPI recommendations to be consistent with the methods used over time. 

**Figure 2:**
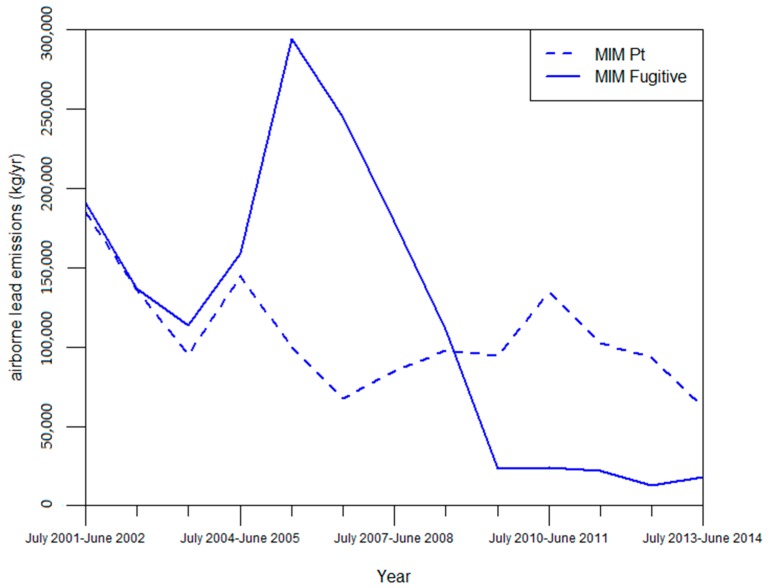
Point and fugitive NPI emission estimates for Mt Isa Mines (MIM) [17].

Temporal comparison of methods to point source emissions shows that despite the fact that methods for estimating fugitive emissions were consistent relative to point sources, fugitive emissions experienced greater year-to-year changes in emissions. Furthermore, substantial changes in emissions often occurred without changes in methods (see Supplementary Materials Table S1). For example, from the year July 2009–June 2010 to July 2010–June 2011, point source emissions increased ~42% without a change in methods. For fugitive emissions, lead emissions from Mt Isa mines dropped ~79% from the year July 2008–June 2009 to July 2009–June 2010, and increased by ~85% from the year July 2005–June 2006 to July 2006–June 2007. On neither occasion did MIM change the methods used to estimate fugitive emissions. Finally, there were also periods of relative consistency, even with method changes, such as for the years of July 2008–June 2009 and July 2009–June 2010 where point source emissions remained at ~95,000 kg (Figure 2). The results suggest that changes in methods may not be the most significant influence on changes of emissions estimates over time, and that additional factors must be accounted for. 

(b) Our analysis found that the methods used over time by the Port Pirie smelter and Broken Hill mine to estimate emissions were relatively consistent for both point source and fugitive emissions. For fugitive source emission estimates, Port Pirie smelter used the same method from the year July 2003–June 2004 to July 2009–June 2010, before adopting an additional method from July 2010-June 2011 onwards. Broken Hill mines used the same method for fugitive emissions estimates every year between the years of July 2001–June 2002 and July 2013–June 2014. For point source emissions estimates, Port Pirie smelter used the same method annually between the years of July 2001–June 2002 and July 2013–June 2014 (except for the year July 2005-June 2006 where an alternative was used).

The relative consistency of methods used by the Port Pirie smelter and Broken Hill mines over time is similar to MIM for estimating fugitive emissions, as all sources had few changes in methods over time. In contrast, the consistency of Port Pirie smelter’s assessment methods for point source emissions differs from MIM, which had frequent method changes and did not consistently use a particular method. These results show that the methods used by MIM are not consistent with similar sources for point source emissions. 

**Figure 3:**
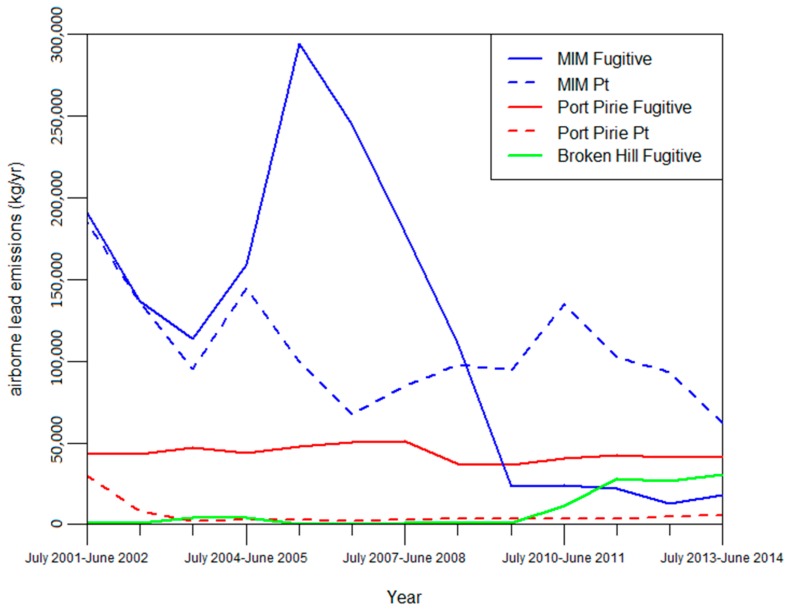
Point source and fugitive NPI emission estimates for Mount Isa Mines, Port Pirie Smelter, and Broken Hill Mines [17].

A clear relationship between method selection and variation in emissions estimates from the three facility sources was not found through temporal comparison. In the case of point source emissions, MIM emissions estimates varied from year-to-year considerably more than the Port Pirie smelter, mirroring the frequent changes to methods used by MIM compared with the Port Pirie smelter as seen in Figure 3. These changes in emissions estimates may be inflated by the quantity of emissions from MIM which were considerably greater than estimates from Port Pirie most years. In contrast, both sources changed methods a similar number of times when estimating fugitive emissions. Furthermore they mostly used the same methods to estimate fugitive emissions. Despite this, fugitive emissions estimates for Port Pirie smelter varied considerably less compared to MIM, remaining consistently around 40,000–50,000 kg/yr. Moreover, the methods used by Broken Hill for fugitive emissions did not change between the years of July 2001–June 2002 and July 2013–June 2014. Yet Broken Hill mine’s fugitive emissions varied considerably more than the Port Pirie smelter. Emissions estimates between the years of July 2005–June 2006 and July 2009–June 2010 remained consistently under 1000 kg before increasing dramatically to ~30,000 kg between July 2011–June 2012 and July 2013–June 2014 (Figure 3). In accordance with the results for MIM, these results suggest that while changes in methods may cause substantial changes in emissions estimation which may render estimates incomparable between sources, they do not correlate enough to be the only factor (see Supplementary Materials Table S1). 

### 3.2. Diffuse Source Emissions

#### 3.2.1. Assessment of Accuracy of Diffuse Source Emissions via Analysis of the Methods Used

Emissions from the Pilbara and Bunbury airsheds were estimated with the formulas listed in the NPI’s EETM for paved/unpaved roads. These methods were mostly sourced from the U.S., including the U.S. EPA AP-42’s handbooks for Unpaved and Paved roads [26]. Analysis of the sources of methods from the EETM, their updates, and external research reveals serious issues regarding outdated values, and considerable evidence of inaccuracy in the formulas applied, as well as several of the values. 

One significant shortcoming in the NPI formulas is the exclusion of average vehicle speed as a significant independent variable. The influence of vehicle speed on emissions shows that there is a significant relationship between vehicle speed and emissions from unpaved roads [27,28,29]. Estimates that do not account for average vehicle speed and average vehicle weight may over (or under) predict emissions from unpaved roads by up to a factor of three [29]. This is significant in the context of the NPI, as emissions from unpaved roads account for ~98% of emissions from paved/unpaved roads for the Pilbara and Bunbury airsheds [30,31]. This research has led to the inclusion of average vehicle speed in the AP-42 formulas for unpaved roads in 2006, but not the averaged weight [32]. These changes are not reflected in the formulas presented in the NPI EETM which still rely on data and methods from the 1970s and 1980s [26,29,30,31,32] (see Supplementary Materials Document S1: Formulas S2 and S3).

Another significant shortcoming in the paved/unpaved roads EETM is the mass speciation values for lead used. Mass speciation values allocate the Total Suspended Particle (TSP) emissions from a diffuse source to specific chemicals to determine the fraction of the total emissions attributed to a particular chemical released. Current mass speciations of chemicals in the paved/unpaved roads EETM are sourced from the California Air Resources Board (CARB) published in 1991 [26]. These, in turn, are from a 1989 study of various soil types in California [33]. These values were measured before the phase-out of leaded petrol. More recent mass speciation datasets based on research in California [34] and across the U.S. for the U.S. EPA’s SPECIATE database [35] show significant decreases in lead on roads, particularly unpaved roads. If more recent lead mass speciations were substituted into the original Bunbury and Pilbara estimates, estimates for lead emissions for paved/unpaved roads would decrease by 84% or 66% if applying the most recent values from CARB and SPECIATE, respectively (see Supplementary Materials Document S1: Table S2). 

Application of any of these datasets is questionable, as they are not based on measurements made in Australia. Nonetheless, these datasets demonstrate that current mass speciation values likely overestimate lead concentrations on paved/unpaved roads due to the phase-out of petrol, significantly contributing to overestimations in lead emissions estimations.

Another significant shortcoming of the paved/unpaved roads EETM is the default values for silt loading. Silt loading is defined as the fraction of road dust which is below 75 μm in diameter [32]. The NPI handbook lists several “default values” for silt loading, which can be used in the absence of locally measured values [26]. These were used to estimate emissions from unpaved and paved roads in the Pilbara and Bunbury airsheds, with a single value allocated to represent a whole airshed. In both the NPI and updated AP-42 formulas, the silt loading of a road is the most influential variable in estimations of emissions [36], so it is important that the value applied for estimating emissions is accurate. The paved/unpaved roads EETM included default values from the 1998 version of the AP-42, which was calculated from 78 samples measured in the 1970s and 1980s [37]. Since then the U.S. National Emission Inventory (NEI) has provided silt content values specifically for each U.S. State for unpaved based on approximately 200 tests with more recent tests recording much lower silt content values [38]. These data resulted in significant decreases in the default values for silt loading applied by the U.S. EPA [39]. Substituting the most recent default value for silt content into the original estimates for the Bunbury airshed would decrease the emissions factor for unpaved roads by 34 percent, demonstrating the significance of these changes in the context of emissions estimates (see Supplementary Materials Document S1: Table S5).

These values may not be transferable to estimates for the Pilbara and Bunbury airsheds due to the high amount of scatter recorded from measurements across the U.S. [38]. Even in the U.S. context, the U.S. EPA does not recommend using default values for variables such as silt loading as they are representative of state or country-wide areas rather than specific airsheds. Instead, it is recommended that locally measured values are used [32,40]. 

Further compounding this problem is the significant spatiotemporal variation in silt loading across large areas. Kavouras et al. [41] shows that the use of a single value to represent a whole airshed is an inaccurate representation of the silt loading across airsheds, and therefore of the total emissions from paved/unpaved roads. Due to the significance of silt loading in the NPI formulas, these issues further demonstrate the magnitude of inaccuracies and uncertainty in the emission estimates for paved/unpaved roads in the Pilbara and Bunbury airsheds.

#### 3.2.2. Assessment of Consistency of Diffuse Source Emissions Data via Comparison of Emissions Estimations from Paved/Unpaved Roads between Airsheds

There are significant differences between the magnitude of the paved/unpaved road emission estimates for WA and other States’ airsheds. As seen in Table 1, the estimated emissions from the Pilbara and Bunbury regions are two orders of magnitude higher than the estimated emissions from any other State. Outside of WA, only Victoria, South Australia, and Tasmania provide any estimates for lead emissions from paved/unpaved roads. 

Comparison of emission estimation methods used by WA and other States demonstrate significant inconsistencies in estimations reported to the NPI. In particular, the methods of accounting for emissions from unpaved roads specifically are a significant source of difference between WA and other states. Emissions from unpaved roads are significant in the context of the Pilbara and Bunbury airsheds, as they account for ~98% of emissions from paved/unpaved roads [30,31]. By contrast, Victoria, South Australia, and Tasmania do not estimate, or significantly reduce estimations for the emissions from unpaved roads due to concerns with overestimation. Victorian diffuse emissions estimates do not estimate emissions from unpaved roads [42]. NPI emissions estimates in Tasmania suggest that this is true there as well (see Supplementary Materials Document S2). The first estimates for diffuse emissions in South Australia made in 2002 did not include unpaved road emissions due to concern with the high degree of uncertainty of the estimates [43]. Furthermore, the most recent update for South Australia which included emissions from unpaved roads reduced the emissions factors to one percent of the value estimated from NPI methods to account for the influence of roughness factors and gravitational dispersion in preventing emissions from travelling regionally [44]. Emissions factors from paved roads were reduced to 15% of their original value for the same reason. 

Another major source of difference between emissions estimates from WA and other States is the mass speciation values used for lead and other chemicals. The mass speciation values applied to the Pilbara and Bunbury airsheds are the default values given in the NPI. By contrast, the emissions estimates for Victoria and South Australia use more recent mass speciation data for lead and other chemicals. In the case of Victoria, mass speciations from the Victoria Air Emissions Inventory, specifically designed for Victorian airborne emissions estimates, were used [42]. This inventory has been updated since its first use for the NPI to use more recent traffic and pollution data in its estimates [42]. In addition, South Australia’s emissions were estimated using the mass speciation values from the 3.2 version of U.S. EPA’s SPECIATE database, updated in 2003. NPI data suggests that the mass speciation values applied by South Australia and Victoria differ substantially from the NPI’s mass speciation values (see Supplementary Materials Document S2). Furthermore, methods in South Australia included revising formulas to account for the AP-42’s 2003 updates of paved and unpaved roads, and included more recent traffic data [44]. The differences in methods used by Victoria, Tasmania, and South Australia explain the substantial differences in emissions between these States and WA. Furthermore, they demonstrate that emissions estimates of paved/unpaved roads between WA and these States are inconsistent.

Analysis of the year of representation of diffuse emissions estimates found that all paved/unpaved roads emissions estimates are not representative of “current” emissions. The webpage for each airshed includes the year that the current emissions estimates are representative of, although it is not clear when using the “Search by Form” tab on the NPI website that diffuse emissions estimates are not re-estimated every year. The figures listed in Table 2 show that for all airsheds, paved/unpaved road emissions are over a decade old. The most recent estimates were made by South Australia in 2003, while all other estimates were made between 1999 and 2003, with the Pilbara and Bunbury airshed estimations representative of the years 2000 and 2003, respectively.

## 4. Discussion

### 4.1. MIM

#### 4.1.1. Accuracy of MIM Airborne Lead Emissions Estimations

The results of the comparison between the average annual lead concentrations measured in Mt Isa with MIM lead emissions estimates from the NPI showed considerable differences. By failing to correlate with external airborne lead measurements, these results suggest that NPI lead emissions estimates do not accurately represent lead emissions from MIM. 

It should be noted that by assessing accuracy through correlation between changes in emissions and concentrations, we assume a consistent relationship between them over time. This would demonstrate that there are inaccuracies present in the estimates, but it would not determine to what extent emissions estimates were inaccurate for each year, since the relationship between emissions and airborne concentration is not known. Therefore we do not know if the most recent 2013–2014 emissions estimates are an accurate representation of emissions from MIM, which it could be argued are of the most interest to users. 

Despite this, there is little to suggest that methods used by MIM to estimate emissions have improved since the July 2009–June 2010 NPI report, as no mention of improved methods has been made in the NPI since the July 2010–June 2011 report. Furthermore, the NPI does not require facility sources to release their calculations for estimating emissions. Irrespective of the improvements made, the legislated goals of the NPI are not specific to the most recent emissions data from facility sources. This suggests that all emissions estimates, not just the most recent, are required by the goals of the NPI to be accurate. This fits with the broader aims of the NPI to provide information to the public and policy makers on the environmental performance of industry sources and the potential risks from emissions. Fulfilling these broader aims requires accurate emissions data over time, as it is hard to determine environmental performance or the success of environmental policy without accurate past emissions data to compare it to. This ultimately prevents such estimates, when proved to have some degree of inaccuracy, from being useable, violating the second legislated goal of the NPI. Our analysis demonstrated that even relatively recent MIM data dating back to the July 2009–June 2010 estimate did not align with the measured airborne lead concentration, ultimately making these data unsuitable for fulfilling the NPI’s broader aims, as well as its legislated goals.

The lack of information provided on estimates made by sources is another serious concern. As a result of the lack of emissions information provided, the NPI’s legislated goal of providing a broad base of emissions information is not fulfilled. Although the methods used are listed in the NPI, the rigour with which these methods or their updates are applied is not known and can greatly affect the accuracy of emissions estimates. The lack of information provided by MIM and by the NPI as a whole, ultimately prevents users from assessing the reliability of emissions, and therefore further inhibits the information necessary for emissions estimates to be useable to all users.

#### 4.1.2. Consistency of MIM with Other Sources 

Our analysis of methods used by MIM to estimate emissions found that methods used to estimate fugitive emissions appeared to be relatively consistent with other major sources, as well as with past emissions estimates. By contrast, the methods used to estimate point source emissions are not consistent over time, as is recommended by the NPI to enable analysis of a facility’s environmental performance over time. Furthermore, they are not consistent with other major facility sources of lead, making comparison with other facilities’ lead emissions misleading for evaluating a source’s environmental performance and potential risk. 

These results suggest that in this case, the NPI fails to provide data that maintain accurate relationships with past estimates and between sources, based on the methods used for point source emissions. This would mean that emissions data from MIM are not understandable to all users and therefore, do not fulfil the NPIs second legislated goal. However the basis of this analysis was the assumption that method changes would influence emissions estimates. Our comparison of emissions estimates with changes in methods over time did not demonstrate this conclusively. Our analysis did show that changes in methods from MIM correlated with variation in emissions estimates relative to the Port Pirie smelter, providing the best evidence that estimates between facility sources were inconsistent as a result of changes in methods. Unfortunately complicating matters further is that the NPI informed us that some of the methods used by MIM to estimate point source emissions as listed in the NPI are incorrect [45]. This could mean that changes in emissions estimates are reflective of MIM’s environmental performance, including attempts to reduce emissions.

According to NPI sources, more recent changes in point source emissions from MIM are due to changes in fuels combusted, volumes of off-gas sent to the facility’s Acid Plant, and the operation of the Air Quality Control Centre (AQCC) which monitors airborne lead levels through several air monitors around Mt Isa [46]. While there is some evidence to suggest this, these claims are hard to verify, as facility sources are not required to release calculations for their emissions. Furthermore, apart from NPI reports submitted by MIM, the main sources of information on fuels combusted and the AQCC are Xstrata sustainability reports which only exist for the years 2007–2011 (see Supplementary Materials Document S3). Irrespective of the validity of these claims, that methods provided by MIM are not accurately listed in the NPI demonstrates a failure of NPI to provide up-to-date information on emissions that can be examined by users to assess its accuracy. This in itself demonstrates a failure of the NPI to fulfil its legislated goal to provide information on emissions that is useful to all users.

Regarding fugitive emissions, while the relationship between fugitive emissions estimates and changes in methods was not demonstrated in our analysis, changes to formulas within a method are responsible for substantial changes in fugitive emissions estimates. NPI sources acknowledge that the drop in fugitive emissions between the years of July 2008–June 2009 and July 2009–June 2010 by ~78% shown in Section 3.1.2 was largely due to updates in the emissions factors for conveying, ore crushing, and the formula for open-air wind erosion [45]. This decrease was followed by four years of consistently low fugitive emissions estimates relative to MIM’s emissions estimates before 2009. In particular the emissions factors for conveying and ore crushing were reduced by over 90% of their previous figure, reducing emissions estimates from these sources considerably. These changes would not be shown in methods and the updates are barely acknowledged in the NPI report for July 2009–June 2010 or in the 2010 Xstrata sustainability report. As a result, emissions estimates give the impression that MIM have drastically reduced their emissions due to improved environmental performance rather than through changes to methods or updates to formulas. Similar changes may have affected point source emissions estimates from MIM as well. 

Changes in the application of formulas for fugitive emissions estimates demonstrates that the information on emissions provided by the NPI is not presented in a form that is consistent with past emissions estimates, or with other sources in the NPI. As user interpretation of NPI data is dependent on maintaining accurate comparisons between sources and past data, our analysis demonstrates that MIM emissions data are not presented in an understandable form to all sections of the community and thus fail to fulfil the NPI’s legislated goals. This is a serious concern as recent studies have shown that there is a high instance of above regulation blood lead levels in Mt Isa which are likely to have been caused by the smelter [19,21]. Our analysis demonstrates that residents in Mt Isa are unable to properly assess the health risks posed by the smelter, due to the inaccuracy and inconsistency of emissions estimates from MIM. Such concerns should be addressed in the NPI review for 2017, as the NPI was designed specifically to provide users with the data to make informed decisions regarding health and environmental risks. 

### 4.2. Paved/Unpaved Roads in Pilbara and Bunbury Airsheds

#### 4.2.1. Accuracy of Paved/Unpaved Roads from Pilbara and Bunbury Airsheds

Analysis of the methods used by the NPI for estimating paved/unpaved road emissions showed several significant sources of inaccuracy. The exclusion of average vehicle weight, the use of mass speciation values for lead, and the use of default values for silt loading, all provide ample evidence of the use of outdated and inaccurate values and formulas used to estimate emissions paved/unpaved roads in the Pilbara and Bunbury airsheds.

The findings of our analysis have serious implications regarding the NPI’s ability to fulfil its legislated goals. The use of outdated data relative to current U.S. sources shows that the NPI is not using the most up-to-date available sources for its emissions estimates of paved/unpaved roads, violating the NPI’s first goal of collecting a broad base of emissions information. The use of outdated and inaccurate formulas and default values provides substantial evidence that estimates for paved/unpaved roads in the Pilbara and Bunbury airsheds are highly inaccurate. This is compounded by the fact that the formulas and data used to estimate paved/unpaved roads were not calculated from data measured in conditions representative of the Pilbara or Bunbury airsheds. The result is that the estimates for the Pilbara and Bunbury regions are highly uncertain, with sufficient evidence of inaccuracy. Our results align with other evidence, such as the absence of lead monitors in WA due to significant decreases in lead measurements after the phase out of leaded petrol [47], suggesting far lower airborne lead emissions than is published in the NPI. This analysis demonstrates that the NPI fails to fulfil its legislated goals which imply the provision of accurate information to users, to enable them to assess potential health and environmental risks that they may be exposed to. By failing to achieve this, the NPI is failing to serve its primary purpose and potentially obscuring significant health risks from Australian communities.

#### 4.2.2. Consistency of Paved/Unpaved Road Estimates from Pilbara/Bunbury Airsheds

The results of our comparisons of methods used to estimate paved/unpaved lead emissions between Pilbara/Bunbury roads and other reporting, shown in Section 3.2.2, found substantial differences between different locations. These differences in methods were responsible for the significantly higher emissions estimates from WA compared to other reporting States. In particular, the reduction or exclusion of unpaved roads emissions estimates is responsible for the substantially lower emissions estimates from other states, as the unpaved roads component accounted for the vast majority of paved/unpaved road emissions from the Pilbara and Bunbury airsheds. Other changes to methods, including mass speciation values changing to updated US values or locally measured and calculated values, reflect a desire to improve on emissions estimates beyond methods offered in the NPI. This becomes a source of inconsistency, as updates are taken at the discretion of the State or Territory authority responsible for estimates and are not coordinated between authorities. More importantly it demonstrates a need for updates to methods provided by the NPI, which are not current or accurate. Without accurate methods to guide diffuse estimations, there is little incentive to update emissions estimates except through seeking methods and research adopted outside the NPI, raising the possibility that different methods will be applied by different States and Territories. This in turn prevents emissions estimates from being comparable to each other, as the relationships between emissions estimated between different States and Territories will not be preserved by NPI data. NPI users seeking to understand the risks posed by emissions from diffuse sources are reliant on the relationship of emissions between sources being conveyed accurately. The failure to preserve these relationships means that paved/unpaved road emissions estimates are not presented in a form that is useful or understandable to users, violating the second legislated goal of the NPI.

This lack of consistency extends beyond diffuse sources. Although our analysis showed that all airsheds that estimated emissions for paved/unpaved roads were representative of roughly consistent years, the nature of the NPI means that comparison of emissions estimates between sources for user-interpretation is likely to extend beyond similar sources to the comparison of diffuse and facility sources. This is problematic as facility sources are required to be updated every year, compared to emissions estimates for paved/unpaved roads which are over 10 years old. This makes them incomparable to facility emissions, potentially distorting user-interpretation of NPI figures for facility sources. As a result, out-dated and inaccurate paved/unpaved roads emissions estimations influence user interpretation of emissions estimates for facility sources, regardless of the accuracy of emissions estimates for those sources. These findings are significant in light of the recently announced NPI review. As this discussion highlights, the current inconsistency of emissions estimates is likely to obscure environmental and health risks—conflicting directly with the stated aims of the NPI. 

## 5. Conclusions

The objective of this study was to assess whether the NPI fulfilled its legislated goals by assessing the accuracy and consistency of emissions estimates from the NPI. This study found that for the largest facility and diffuse sources of lead emissions, Mt Isa Mines and paved/unpaved roads in the Pilbara and Bunbury airsheds, emissions estimates in the NPI were not accurate and were inconsistent with past data and other sources. As a result emissions figures from these sources do not fulfil the goals of the NPI to provide emissions information that is accurate, up-to-date, and understandable to all sectors of the community.

The significance of the sources assessed in this study are likely to be of wide-spread interest to non-scientifically trained users and as such, may be misleading to many users who wish to examine the environmental performance and health risks posed by industries that the NPI was designed to make accessible. At a minimum, the upcoming NPI review must address the issues found in this paper and investigate whether these issues are present for other source types and chemicals examined within the NPI. 

## Figures and Tables

**Table 1:** Airborne lead emissions from Paved and Unpaved roads combined for the disaggregated airsheds of Bunbury and Pilbara, WA and all States and Territories for 2013–2014 (rounded to the nearest thousand) [17].

Airshed/State or Territory	Paved/Unpaved Lead Emissions Listed in the NPI (kg/yr)
Bunbury (WA)	382,000
Pilbara (WA)	125,000
Victoria	8900
South Australia	2400 ^1^
Tasmania	1400
Queensland	Not listed
New South Wales	Not listed
Australian Capital Territory	Not listed
Northern Territory	Not listed

NPI: National Pollutant Inventory. ^1^ Total South Australia estimates for lead from paved/unpaved roads are listed in the NPI as 2700 kg/yr. However, 11 of the airsheds exist within six major airsheds within South Australia. The figure shown has been adjusted to avoid double-counting by only summing the emissions from the major airsheds.

**Table 2:** Year of representation for current NPI estimates of paved/unpaved roads [17].

State	Airshed	Year of Representation
Western Australia	Bunbury	2003
	Pilbara	2000
South Australia ¹	Adelaide	2003
	Barmera	2003
	Barossa	2003
	Berri	2003
	Loxton	2003
	Lyndoch	2003
	Milicent	2003
	Mt Gambier	2003
	Nuriootpa	2003
	Port Augusta	2003
	Port Lincoln	2003
	Port Pirie	2003
	Renmark	2003
	Riverland	2003
	South East	2003
	Spencer Gulf	2003
	Whyalla	2003
Victoria	Ballarat Region	2002
	Bendigo Region	2001
	Latrobe Valley Region	2000
	Mildura Region	2002
	Port Philip Region	1999
Tasmania	Hobart	1999
	Launceston	2000

^1^ The figures for the year of representation for airsheds from South Australia are different in the NPI, which lists that all airsheds except for Adelaide were representative of the year 2000. However, according to South Australia’s Environmental Protection Authority [44] and a review of the NPI [6], these airsheds were all updated to be representative of emissions from 2003.

## Supplementary Materials

The following are available online at www.mdpi.com/1660-4601/14/5/478/s1, Figure S1: Location of greatest sources of airborne lead emissions in the 2013/14 NPI report, Figure S2: Location of MIM smelters and acid plant, and the EHP air quality monitoring station in Mt Isa, Qld, Table S1: Methods of estimation used by Mount Isa Mines, Port Pirie Smelter and Broken Hill Mines for point and fugitive emission estimations. Document S1: Comparison of formulas and default values used to estimate emissions from paved and unpaved roads, which includes, Table S2: Mass speciation values for lead from paved and unpaved roads as listed by the NPI, CARB and U.S. EPA, with equivalent estimates for lead emissions from paved/unpaved roads in the Pilbara and Bunbury airsheds if all other formulas and values were consistent with the original methods used, Table S3: Default silt loading fractions for unpaved roads used by the U.S. EPA, Table S4: Default silt loading fractions for unpaved roads used by the NPI, Table S5: Estimates for lead emissions from unpaved roads in the Pilbara and Bunbury airsheds for default silt loading values from NPI and U.S. EPA if formulas and all other values were consistent with the original methods used, Table S6: Default silt loading fractions for paved roads used by the U.S. EPA, Table S7: Default silt loading fractions for paved roads used by the NPI. Formula S1: Formula for estimating emissions from paved or unpaved roads as used by U.S. EPA and NPI, Formula S2: Formula for estimating the emission factor for Total Suspended Particulate (TSP) emissions from unpaved roads used by U.S. EPA, Formula S3: Formula for estimating the emission factor for TSP emissions from unpaved roads used by NPI, Formula S4: Formula for estimating the emission factor for TSP emissions from paved roads used by U.S. EPA, Formula S5: Formula for estimating the emission factor for TSP emissions from paved roads used by NPI. Document S2: Mass speciation values and emissions estimates for various chemicals relative to lead, which includes: Table S8: Paved and Unpaved road mass speciation values for lead, cobalt, copper, manganese and zinc and their ratio relative to lead mass speciation from the paved/unpaved roads in NPI EETM, Table S9: Paved/Unpaved road emissions estimates for lead, cobalt, copper, manganese and zinc and their ratio of emissions relative to lead emissions/mass speciation from the Launcheston and Hobart airsheds, Tasmania, Table S10: Paved/Unpaved road emissions estimates for lead, cobalt, copper, manganese and zinc and their ratio of emissions relative to lead emissions from the Port Philip Region (PPR) airshed, Victoria, Table S11: Paved/Unpaved road emissions estimates for lead, cobalt, copper, manganese and zinc and their ratio of emissions relative to lead emissions from the Adelaide airshed, South Australia. Document S3: Variables influencing airborne emissions from MIM, which includes, Table S12: Number of hours the lead and copper smelters at MIM were either on total or partial shutdown each year, Table S13: Total material usage of diesel, unleaded fuel, LPG and Wood from MIM from 2007 to 2011, Table S14: Amount of lead bullion mined per year at Mt Isa Mines.

## Appendix A. Methods for Estimating Average Annual Airborne Lead Concentrations from EHP Data

To estimate the annual average values for EHP measurements for all years between July 2009–June 2010 and July 2013–June 2014, we used four different measures to account for a number of values that were recorded below a detection limit; the Kaplan-Meier (K-M) method, Lognormal robust Regression on Order Statistics (LROS), the maximum possible value, and the minimum possible value.

The Kaplan-Meier method is a non-parametric measure used to estimate summary statistics for datasets with censored values, such as values below a detection limit, and which makes no assumptions on the distributional shape of data [48]. This was useful for our dataset, as it was difficult to determine the distribution of values below a detection limit. The only disadvantage to the Kaplan-Meier method is that it is susceptible to over-estimating averages when the lowest recorded value is below a detection limit [48,49]. This was true for all our data, particularly the year July 2009 to June 2010 where half the values were below the same detection limit. In these cases, K-M estimates tend to be very close to the maximum possible value.

We supplemented the results of the Kaplan-Meier analysis by using LROS to estimate annual average values for the EHP measurements. LROS imputes values for non-detect values to a lognormal distribution, which is assumed to follow the distribution of the detected values [48]. This method is less likely to overestimate the annual average, even when the lowest recorded value was below a detection limit. The main disadvantage of LROS is that we could not prove that a log-normal distribution was followed by values below the detection limits. Goodness-of-Fit tests performed on the dataset to determine the appropriate distribution of EHP values found that there was significant uncertainty about the distribution of values under a detection limit, due to the large positive skew of the data.

To supplement the estimates from the K-M method and LROS, the minimum possible and maximum possible yearly averages were calculated to show the potential error of both trends. The minimum possible yearly average was calculated by substituting censored values with zero values, while the maximum possible yearly average was calculated by substituting each censored value with its detection limit.

## Appendix B. Methods Used by Point Sources to Estimate Airborne Lead Emissions

The NPI lists four different methods for industry to estimate point source emissions, which are defined as those that “flow into a vent or stack and are emitted through a single point source”, and fugitive emissions, defined as those that “are not released via a vent or stack” [50] (p. 20). These methods are: mass balance estimation, engineering calculations estimation, direct measurement estimation, and emission factor estimation. A company uses at least one of these methods when estimating emissions for individual chemicals, or alternatively, a company may apply to have an alternative method approved through the NPI.

## References

[1] National Environment Protection Council (1998). National Environment Protection (National Pollutant Inventory) Measure 1998.

[2] Howes M. (2001). What’s your poison? The Australian national pollutant inventory versus the U.S. toxics release inventory. Aust. J. Political Sci..

[3] National Pollutant Inventory Technical Advisory Panel (2006). Final Report to the National Environment Protection Council.

[4] Streets S., Di Carlo A. (1999). Australia’s first national environmental protection measures: Are we advancing, retreating or simply marking time?. Environ. Plan. Law J..

[5] Sullivan R. (1999). The national environment protection measure for the national pollutant inventory: Legal, technical and policy issues. Environ. Plan. Law J..

[6] Department of the Environment and Heritage (2005). Review of the National Pollutant Inventory for the Department of the Environment and Heritage.

[7] Commonwealth Environmental Protection Agency (1994). National Pollutant Inventory: Public Discussion Paper.

[8] National Toxics Network Inc. Australia’s national pollutant inventory—Has it served community right to know?. Proceedings of the Pollutant Inventory Conference.

[9] Deegan C., Rankin M. (1999). The environmental reporting expectations gap: Australian evidence. Br. Account. Rev..

[10] U.N. Environment Programme (1992). Rio Declaration on Environment and Development. http://www.unep.org/documents.multilingual/default.asp?documentid=78&articleid=1163.

[11] Organisation for Economic Co-operation and Development (1996). Pollutant Release and Transfer Registers (PRTRs): A Tool for Environmental Policy and Sustainable Development.

[12] Organisation for Economic Co-operation and Development (1996). Recommendation of the Council on Implementing Pollutant Release and Transfer Registers.

[13] Ellson A., Johnston D. (2005). Interpretive Guide for the NPI-A guide to understanding South Australia’s NPI Data.

[14] Burritt R., Saka C., Schaltegger S., Bennett M., Burritt R. (2006). Quality of physical environmental management accounting information: Lessons from pollutant release and transfer registers. Sustainability Accounting and Reporting.

[15] Cowan S., Deegan C. (2011). Corporate disclosure reactions to Australia’s first national emission reporting scheme. Account. Financ..

[16] National Environment Protection Council (2016). Review of the National Pollutant Inventory: Terms of Reference, November 2016.

[17] National Pollutant Inventory NPI Data. http://www.npi.gov.au/npi-data/search-npi-data.

[18] Marchi S.D., Hamilton J.T. (2006). Assessing the accuracy of self-reported data: An evaluation of the toxics release inventory. J. Risk Uncertain..

[19] Mackay A.K., Taylor M.P., Munksgaard N.C., Hudson-Edwards K.A., Burn-Nunes L. (2013). Identification of environmental lead sources and pathways in a mining and smelting town: Mount Isa, Australia. Environ. Pollut..

[20] Taylor M.P., Davies P.J., Kristensen L.J., Csavina J.L. (2014). Licenced to pollute but not to poison: The ineffectiveness of regulatory authorities at protecting public health from atmospheric arsenic, lead and other contaminants resulting from mining and smelting operations. Aeolian Res..

[21] Noller B., Zheng J., Huynh T., Ng J., Diacomanolis V., Taga R., Harris H. (2017). Lead Pathways Study—Air: Health Risk Assessment of Contaminants to Mount Isa City.

[22] Csavina J., Field J., Taylor M.P., Gao S., Landázuri A., Betterton E.A., Sáez A.E. (2012). A review on the importance of metals and metalloids in atmospheric dust and aerosol from mining operations. Sci. Total Environ..

[23] Department of Environment and Heritage Protection Air Reports and Plans. https://www.qld.gov.au/environment/pollution/monitoring/air-reports/.

[24] Department of Environment and Heritage Protection Hourly Air Quality Data. http://www.ehp.qld.gov.au/air/data/search.php.

[25] Department of Environment Regulation (2015). 2014 Western Australia Air monitoring Report.

[26] National Pollutant Inventory (1999). Emissions Estimation Technique Manual for Aggregated Emissions from Paved and Unpaved Roads.

[27] Gillies J.A., Etyemezian V., Kuhns H., Nikolic D., Gillette D.A. (2005). Effect of vehicle characteristics on unpaved road dust emissions. Atmos. Environ..

[28] Gillies J., Arnott W.P., Etyemezian V., Kuhns H., Mossmüller H., DuBois D., Abu-Allaban M. (2005). Characterizing and Quantifying Local and Regional Particulate Matter Emissions from Department of Defense Installations.

[29] Kuhns H., Gillies J., Etyemezian V., Nikolich G., King J., Zhu D., Uppapalli S., Engelbrecht J., Kohl S. (2010). Effect of soil type and momentum on unpaved road particulate matter emissions from wheeled and tracked vehicles. Aerosol Sci. Technol..

[30] Sinclair Knight Merz (2003). Aggregated Emissions Inventory of NPI Substances for the Bunbury Regional Airshed.

[31] Sinclair Knight Merz (2003). Aggregated Emissions Inventory for the Pilbara Airshed 1999/2000.

[32] U.S. Environmental Protection Agency (2006). AP-42, Fifth Edition Compilation of Air Pollutant Emission Factors, Volume 1: Stationary Point and Area Sources.

[33] Houck J.E., Chow J.C., Watson J.G., Simons C.A., Pritchett L.C., Goulet J.M., Frazier C.A. (1989). Determination of Particle Size Distribution and Chemical Composition of Particulate Matter from Selected Sources in California.

[34] Chow J.C., Watson J.G., Ashbaugh L.L., Magliano K.L. (2003). Similarities and differences in PM_10_ chemical source profiles for geological dust from the San Joaquin Valley, California. Atmos. Environ..

[35] Reff A., Bhave P.V., Simon H., Pace T.G., Pouliot G.A., Mobley J.D., Houyoux M. (2009). Emissions inventory of PM_2.5_ trace elements across the United States. Environ. Sci. Technol..

[36] Tong X., Luke E.A., Smith R. (2014). Numerical validation of a near-Field fugitive dust model for vehicles moving on unpaved surfaces. Proc. Inst. Mech. Eng. Part D J. Automob. Eng..

[37] Midwest Research Institute (1998). Emission Factor Documentation for AP-42 Section 13.2.2—Unpaved Roads. AP-42, Fifth Edition Compilation of Air Pollutant Emission Factors, Volume 1: Stationary Point and Area Sources.

[38] Sinclair Knight Merz (2005). Improvement of NPI Fugitive Particulate Matter Emission Estimation Rechniques.

[39] U.S. Environmental Protection Agency Unpaved Road Surface Material Silt Content Values Used in the 1999 NEI. https://www3.epa.gov/ttn/chief/ap42/ch13/related/c13s02–2.html.

[40] U.S. Environmental Protection Agency (2011). AP-42, Fifth Edition Compilation of Air Pollutant Emission Factors, Volume 1: Stationary Point and Area Sources.

[41] Kavouras I.G., DuBois D.W., Nikolich G., Corral Avittia A.Y., Etyemezian V. (2016). Particulate dust emission factors from unpaved roads in the U.S. Mexico border semi-arid region. J. Arid Environ..

[42] Delaney W., Marshall A. Victorian air emissions inventory for 2006. Proceedings of the Clean Air Society of Australia and New Zealand.

[43] Ciuk J. (2002). South Australian NPI Summary Report: Adelaide and Regional Airsheds Air Emissions Study 1998–1999.

[44] Ng Y.L., Johnston D. (2007). Update of South Australia’s National Pollutant Inventory: Aggregate Emissions Data 2002–2003.

[45] Mee L. (2016). Personal communication.

[46] Xstrata Mt Isa Mines (2011). Xstrata Mount Isa Mines: Sustainability Report 2011.

[47] Department of Environment and Conservation (2009). 2008 Western Australia Air Monitoring Report.

[48] Helsel D.R. (2011). Statistics for Censored Environmental Data Using Minitab^®^ and R.

[49] Helsel D.R. (2005). Non-Detects and Data Analysis: Statistics for Censored Environmental Data.

[50] National Pollutant Inventory (2015). National Pollutant Inventory Guide—Version 6.1.

