# Supplementary material for: The Australian National Pollutant Inventory Fails to Fulfil Its Legislated Goals
Source: Int J Environ Res Public Health. 2017 May 4;14(5):478. doi: 10.3390/ijerph14050478 (PMC5451929; doi:10.3390/ijerph14050478)
Supplement: Supplementary file 1 [file ijerph-14-00478-s001.pdf]

Supplementary Materials: The Australian National Pollutant Inventory Fails to Fulfil Its Legislated Goals

Nathan Cooper, Donna Green and Katrin J. Meissner

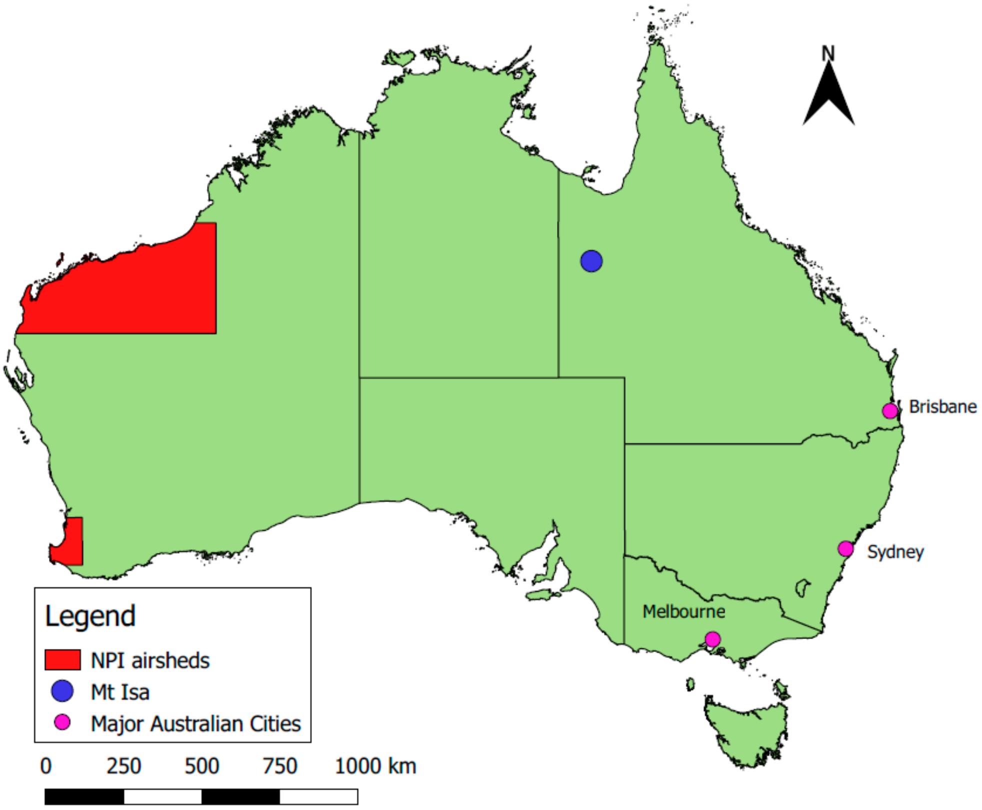

Figure S1. Location of greatest sources of airborne lead emissions in the 2013/14 NPI report [1-3].

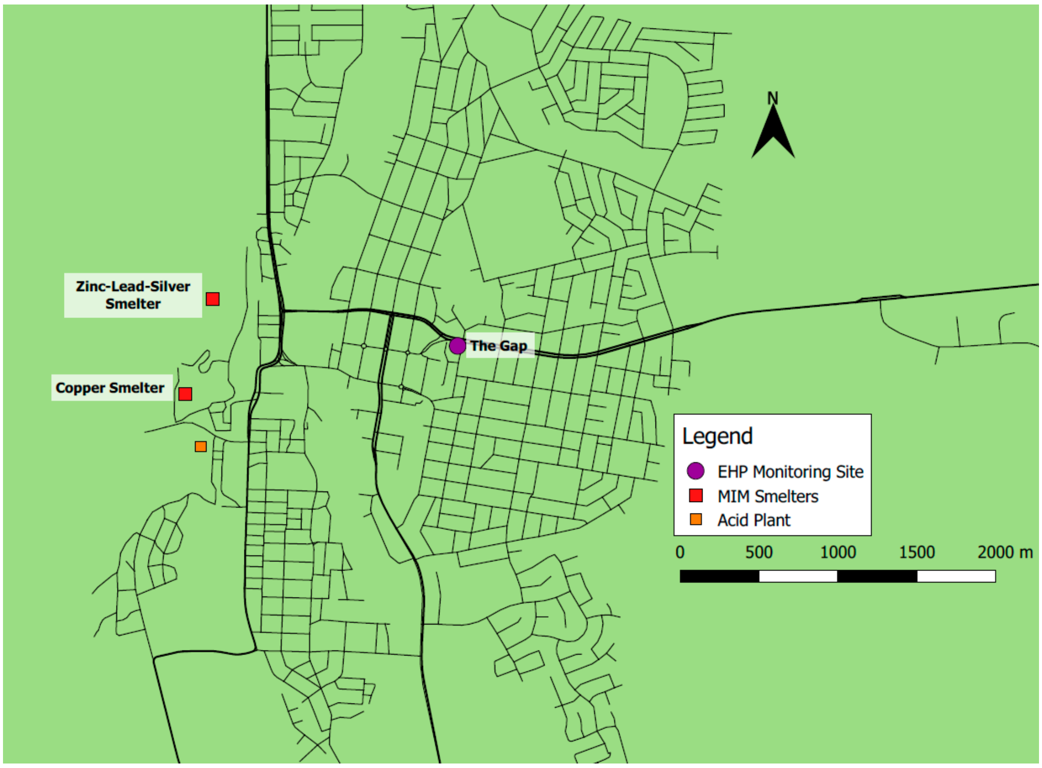

Figure S2. Location of MIM smelters and acid plant, and the EHP air quality monitoring station in Mt Isa, Qld [4].

Table S1. Methods of estimation used by Mount Isa Mines, Port Pirie Smelter and Broken Hill Mines for point and fugitive emission estimations [5].

NPI source	7/2001-6/2002	7/2002-6/2003	7/2003-6/2004	7/2004-6/2005	7/2005-6/2006	7/2006-6/2007	7/2007-6/2008	7/2008-6/2009	7/2009-6/2010	7/2010-6/2011	7/2011-6/2012	7/2012-6/2013	7/2013-6/2014
MIM Pt	Mass Balance; Emission Factors	Mass Balance; Engineering Calculations; Direct Measurement; Emission Factors	Mass Balance; Engineering Calculations; Direct Measurement; Emission Factors	Emission Factors	Emission Factors	Mass Balance; Direct Measurement	Engineering Calculations; Emission Factors	Mass Balance; Direct Measurement; Emission Factors	Mass Balance; Direct Measurement; Emission Factors	Mass Balance; Direct Measurement; Emission Factors	Mass Balance	Mass Balance	Engineering Calculations
MIM Fugitive	Emission Factors	Emission Factors	Emission Factors	Emission Factors	Emission Factors	Emission Factors	Engineering Calculations; Emission Factors	Direct Measurement; Emission Factors	Direct Measurement; Emission Factors	Direct Measurement; Emission Factors	Emission Factors	Emission Factors	Emission Factors
Port Pirie Pt	Direct Measurement	Direct Measurement	Direct Measurement	Direct Measurement	Approved Alternative	Direct Measurement	Direct Measurement	Direct Measurement	Direct Measurement	Direct Measurement	Direct Measurement	Direct Measurement	Direct Measurement
Port Pirie Fugitive	Emission Factors	Direct Measurement; Emission Factors	Emission Factors	Emission Factors	Emission Factors	Emission Factors	Emission Factors	Emission Factors	Emission Factors	Emission Factors; Approved Alternative	Approved Alternative	Approved Alternative	Approved Alternative
Broken Hill Fugitive	Emission Factors	Emission Factors	Emission Factors	Emission Factors	Emission Factors	Emission Factors	Emission Factors	Emission Factors	Emission Factors	Emission Factors	Emission Factors	Emission Factors	Emission Factors

Method	
Mass Balance Estimation	
Engineering Calculations Estimation	
Direct Measurement Estimation	
Emission Factors Estimation	
Approved Alternative Estimation	

Document S1. Comparison of Formulas and Default Values Used to Estimate Emissions from Paved and Unpaved Roads

Mass Speciation

Table S2. Mass speciation values for lead from paved and unpaved roads as listed by the NPI [1,2,6], CARB [7] and U.S. EPA [8,9], with equivalent estimates for lead emissions from paved/unpaved roads in the Pilbara and Bunbury airsheds if all other formulas and values were consistent with the original methods used (3 significant places).

Source	Paved (%)	Unpaved (%)	Pilbara (kg/yr)	Bunbury (kg/yr)
NPI (original estimates)	0.0951	0.0867	125000	382000
CARB	0.0124	0.0130	18700	57100
U.S. EPA	0.0667	0.0288	42900	130000

Silt Loading Default Values

Unpaved Roads

Each U.S. State that has had silt loading samples collected and tested have their own unique State-wide silt loading default value in the absence of more locally attained data. Otherwise States are offered a more general default value listed below

Table S3. Default silt loading fractions for unpaved roads used by the U.S. EPA [10].

Default value	Average value	Highest value	Lowest value
3.9%	3.8%	7.2%	1.5%

Table S4. Default silt loading fractions for unpaved roads used by the NPI [6].

Gravel roads	Dirt roads
6.4%	11%

Table S5. Estimates for lead emissions from unpaved roads in the Pilbara and Bunbury airsheds for default silt loading values from NPI [6] and U.S. EPA [10] if formulas and all other values were consistent with the original methods used (3 significant places).

	Bunbury	Pilbara
NPI original silt value applied	6.4%	11%
NPI original emissions estimate	374000 kg/yr	122000 kg/yr
U.S. EPA equivalent default silt value	3.9% (default)	7.2% (highest)
U.S. EPA equivalent emissions estimate	251000 kg/yr	86900 kg/yr

Paved Roads

Default silt loading values for paved roads are determined by the average daily traffic (ADT) measured on a paved road, as well as the conditions of paved roads [6,11].

Table S6. Default silt loading fractions for paved roads used by the U.S. EPA [11].

ADT category	<500	500–5000	5000–10000	>10000
Ubiquitous baseline	0.6 g/m²	0.2 g/m²	0.06 g/m²	0.03 g/m², 0.015 g/m² limited access
Ubiquitous Winter baseline, during months with frozen precipitation	2.4 g/m²	0.6 g/m²	0.12 g/m²	0.03 g/m², 0.015 g/m² limited access
Initial peak additive contribution from application of antiskid abrasive	2 g/m²	2 g/m²	2 g/m²	2 g/m²
Days to return to baseline conditions	7	3	1	0.5

Table S7. Default silt loading fractions for paved roads used by the NPI [6].

	High ADT Roads (>5000)	Low ADT Roads (<5000)
Normal conditions	0.1 g/m²	0.4 g/m²
Worst case conditions	0.5 g/m²	3 g/m²

Emissions Factor Formulas

All formulas for paved and unpaved roads as shown below estimate the total emissions by multiplying the calculated emissions factor with the total vehicle distance travelled. This product is multiplied to the mass speciation value of a specific chemical to estimate the emissions for that chemical (for example lead). For U.S. formulas, additional measures are applied to convert estimates from pounds to kilograms.

$$E_i = EF \times VDT \times m_i \tag{1}$$

Formula S1. Formula for estimating emissions from paved or unpaved roads as used by U.S. EPA and NPI.

E = Total airshed emissions of chemical *i* from paved or unpaved roads (kg/yr)
EF = Emission factor for paved or unpaved roads (kg/km or kg/mi)
VDT = Total Vehicle Distance Travelled in airshed (km/yr or mi/yr)
m = mass speciation value for chemical *i* (%)

Unpaved roads

$$EF = \frac{k(\frac{s}{12})(\frac{S}{30})^{0.3}}{(\frac{M}{0.5})^{0.3}} - C \tag{2}$$

Formula S2. Formula for estimating the emission factor for Total Suspended Particulate (TSP) emissions from unpaved roads used by U.S. EPA [12].

EF = emission factor (lb/mi)
k = empirical constant (lb/mi) (6.0 for TSP)
s = surface material silt content (%)
S = mean vehicle speed (mph)
M = surface material moisture content (%)
C = emissions factor for 1980's vehicle fleet exhaust, brake wear and tire wear (lb/mi)

$$EF = \frac{k(\frac{s}{12})^{0.8}(\frac{AW}{3})^{0.5}}{(\frac{M}{0.2})^{0.4}} \tag{3}$$

Formula S3. Formula for estimating the emission factor for TSP emissions from unpaved roads used by NPI [6].

EF = Emission Factor (kg/VKT)
k = empirical constant (kg/VKT) (2.82 for TSP)
s = surface material silt content (%)
AW = average weight of vehicles (tonnes)
M = surface material moisture content (%)

Paved roads

$$EF = k(s)^{0.91} \times (AW)^{1.02} \quad (4)$$

Formula S4. Formula for estimating the emission factor for TSP emissions from paved roads used by U.S. EPA [11].

EF = Emission Factor (lb/mi)
k = empirical constant (lb/mi) (0.011 for TSP)
s = surface material silt content (g/m²)
AW = average weight of vehicles (tonnes)

$$EF = k(\frac{s}{2})^{0.65} \times (\frac{AW}{3})^{1.5} \quad (5)$$

Formula S5. Formula for estimating the emission factor for TSP emissions from paved roads used by NPI [6].

EF = Emission Factor (kg/km)
k = empirical constant (kg/km) (0.024 for TSP)
s = surface material silt content (g/m²)
AW = average weight of vehicles (tonnes)

Document S2. Mass Speciation Values and Emissions Estimates for Various Chemicals Relative to Lead

Table S8. Paved and Unpaved road mass speciation values for lead, cobalt, copper, manganese and zinc and their ratio relative to lead mass speciation from the paved/unpaved roads in NPI EETM [6].

**Source**	**Mass Speciation Values (%)**					**Mass Speciation Relative to Lead**			
	Pb	Co	Cu	Mg	Zn	Co/Pb	Cu/Pb	Mg/Pb	Zn/Pb
Paved	0.0951	0.0116	0.0161	0.0795	0.0936	0.12198	0.16930	0.83596	0.98423
Unpaved	0.0867	0.0143	0.0088	0.0973	0.0605	0.16494	0.10150	1.12226	0.69781

Tasmania

Table S9. Paved/Unpaved road emissions estimates for lead, cobalt, copper, manganese and zinc and their ratio of emissions relative to lead emissions/mass speciation from the Launcheston and Hobart airsheds, Tasmania [5].

**Source**	**Emissions Estimates (kg/yr)**					**Emissions Relative to Lead**			
	Pb	Co	Cu	Mg	Zn	Co/Pb	Cu/Pb	Mg/Pb	Zn/Pb
Launcheston	491.96	59.96	82.86	410.99	484.14	0.12188	0.16843	0.83541	0.98409
Hobart	949.59	115.80	160.76	793.76	934.65	0.12195	0.16930	0.83589	0.98427

Victoria

Table S10. Paved/Unpaved road emissions estimates for lead, cobalt, copper, manganese and zinc and their ratio of emissions relative to lead emissions from the Port Philip Region (PPR) airshed, Victoria [5].

Source	Emissions Estimates (kg/yr)					Emissions Relative to LEAD			
	Pb	Co	Cu	Mg	Zn	Co/Pb	Cu/Pb	Mg/Pb	Zn/Pb
PPR	8,490.8	0	2,280.8	2,280.8	654.88	0	0.268617	0.268617	0.07713

South Australia

Table S11. Paved/Unpaved road emissions estimates for lead, cobalt, copper, manganese and zinc and their ratio of emissions relative to lead emissions from the Adelaide airshed, South Australia [5].

Source	Emissions Estimates (kg/yr)					Emissions Relative to Lead			
	Pb	Co	Cu	Mg	Zn	Co/Pb	Cu/Pb	Mg/Pb	Zn/Pb
Adelaide	1,273.0	0	2,615.4	711.35	1,064.9	0	2.054576	0.558815	0.836572

Document S3 Variables Influencing Airborne Emissions from MIM

Air Quality Control Centre (AQCC)

The AQCC has an alert process when weather conditions are likely to cause hazardous levels of airborne lead, sulphur dioxide and other chemicals [13]. The AQCC alert process has four stages: unrestricted operations, standby, reduced operations (partial shutdown) and total shutdown. Data from XMIM listing the number of hours that MIM operations were halted do not differentiate between total and partial shutdown [3,14–18]. Xstrata's 2012 report for North Queensland acknowledged that the number of hours the copper smelter had been partially or totally shutdown for was exaggerated by including hours on standby, with the corrected number of hours in brackets. It is not known whether this was the case for the copper smelter for previous years, or for the lead smelter as Xstrata's 2012 report only reported on Xstrata's copper operations. The figure for 2015 comes from the Mt Isa Mines website, and did not differentiate between the lead and copper smelters [19].

Table S12. Number of hours the lead and copper smelters at MIM were either on total or partial shutdown each year.

Year	Lead Smelter (hrs)	Copper Smelter (hrs)	Total (hrs)
2006	200	654	854
2007	294	1050	1344
2008	385	1021	1406
2009	482	931	1413
2010	536	889	1425
2011	607	868 (316)	1475 (923)
2012	NA	787	NA
2015	NA	NA	1829

Fuels Combusted

Table S13. Total material usage of diesel, unleaded fuel, LPG and Wood from MIM from 2007–2011 [46, 57-60].

Year	Diesel (kL)	Unleaded (kL)	LPG (kL)	Wood (t)
2007	45,655	138	343	1,867
2008	60,283.85	89.58	65	120
2009	71,430	167	525	206
2010	51,602	278	578	278

2011	62,334	97	337	321

Lead Production

Lead production figures came from XMIM sustainability reports [46, 57-60] and Glencore Production Reports [20-22]. For the year 2011, different figures for lead production were given by the annual Glencore production report and the Xstrata sustainability report. Both are included in Table S14, with the figure from the Xstrata sustainability report included in brackets.

Table S14. Amount of lead bullion mined per year at Mt Isa Mines.

Year	Lead in Bullion (Mt)	Zinc-Lead Ore Mined (Million t)
2007	125.2	5.12
2008	166.9	6.4
2009	146.0	7.4
2010	140.1	8.6
2011	130.7 (138.7)	9.1
2012	153.1	NA
2013	167.8	NA
2014	170.2	NA

References

1. Sinclair Knight Merz. *Aggregated Emissions Inventory of NPI Substances for the Bunbury Regional Airshed*; Sinclair Knight Merz: Perth, Australia, 2003.
2. Sinclair Knight Merz. *Aggregated Emissions Inventory for the Pilbara Airshed 1999/2000*; Sinclair Knight Merz: Perth, Australia, 2003.
3. Xstrata Mt Isa Mines. *Xstrata Mount Isa Mines: Sustainability Report 2011*; Xstrata Mt Isa Mines: Brisbane, QLD, 2011.
4. Department of Science, Information Technology, Innovation and the Arts. *Air Quality Bulletin—North Queensland July 2014*; Department of Science, Information Technology, Innovation and the Arts: Brisbane, Australia, 2014.
5. National Pollutant Inventory. NPI data. Available online: http://www.npi.gov.au/npi-data/search-npi-data (accessed on 17 December 2016). Data published and made available under a Creative Commons Attribution 4.0 International Public Licence: https://creativecommons.org/licenses/by/4.0/legalcode
6. National Pollutant Inventory. *Emissions Estimation Technique Manual for Aggregated Emissions from Paved and Unpaved Roads*; Environment Australia: Canberra, 1999.
7. California Air Resources Board. Paved and Unpaved Road Dust PM Profiles. Available online: https://www.arb.ca.gov/ei/speciate/roaddust.htm (accessed on 8 February 2016).
8. United States Environmental Protection Agency. SPECIATE Data Browser: Unpaved Road Dust-Composite. Available online: https://cfpub.epa.gov/si/speciate/ (accessed on 15 February 2016).
9. United States Environmental Protection Agency. SPECIATE Data Browser: Paved Road Dust-Composite. Available online: https://cfpub.epa.gov/si/speciate/ (accessed on 15 February 2016).
10. U.S. Environmental Protection Agency. Unpaved Road Surface Material Silt Content Values Used in the 1999 NEI. Available online: https://www3.epa.gov/ttn/chief/ap42/ch13/related/c13s02-2.html (accessed on 15 February 2016).
11. U.S. Environmental Protection Agency. *Fifth Edition Compilation of Air Pollutant Emission Factors, Volume 1: Stationary Point and Area Sources*; U.S. Environmental Protection Agency: Washington, DC, USA, 2011.
12. U.S. Environmental Protection Agency. *Fifth Edition Compilation of Air Pollutant Emission Factors, Volume 1: Stationary Point and Area Sources*, U.S. Environmental Protection Agency: Washington, DC, USA, 2006.
13. Mount Isa Mines. Air Quality in Mt Isa: Community information about sulphur dioxide management at Mount Isa Mines, Mount Isa Mines: Mount Isa, Australia, 2016.

14. Xstrata. *Xstrata Cooper North Queensland Operations: Sustainability Report 2012*, Xstrata: Brisbane, Australia, 2012.
15. Xstrata Mount Isa Mines. *Xstrata Mount Isa Mines: Sustainability Report 2010*, Xstrata Mount Isa Mines: Brisbane, Australia, 2010.
16. Xstrata Mount Isa Mines. *Xstrata Mount Isa Mines: Sustainability Report 2009*, Xstrata Mount Isa Mines: Brisbane, Australia, 2009.
17. Xstrata Mount Isa Mines. *Xstrata Mount Isa Mines: Sustainability Report 2008*, Xstrata Mount Isa Mines: Brisbane, Australia, 2008.
18. Xstrata Mount Isa Mines. *Xstrata Mount Isa Mines: Sustainability Report 2007*, Xstrata Mount Isa Mines: Brisbane, Australia, 2007.
19. Mount Isa Mines 2016. Air Quality in Mt Isa Portal. Available online: http://www.mountisamines.com.au/EN/sustainability/Pages/AirqualityinMountIsa.aspx (accessed on 26 April 2016).
20. Glencore. *Production Report for the 12 months ended 31 December 2014*; Glencore: Baar, Switzerland, 2015.
21. GlencoreXstrata. *Production Report for the 12 months ended 31 December 2013*; GlencoreXstrata: Baar, Switzerland, 2014.
22. Glencore. *Production Report for the 12 months ended 31 December 2012*; Glencore: Baar, Switzerland, 2013.

© 2017 by the authors; licensee MDPI, Basel, Switzerland. This article is an open access article distributed under the terms and conditions of the Creative Commons by Attribution (CC-BY) license (http://creativecommons.org/licenses/by/4.0/).
